# Supplementary material for: Inhibition of IFNAR-JAK signaling enhances tolerability and transgene expression of systemic non-viral DNA delivery
Source: Mol Ther Nucleic Acids. 2025 Mar 5;36(2):102502. doi: 10.1016/j.omtn.2025.102502 (PMC11979999; doi:10.1016/j.omtn.2025.102502)
Supplement: Document S1. Figures S1 and S2 [file mmc1.pdf]

OMTN, Volume *36*

Supplemental information

Inhibition of IFNAR-JAK signaling enhances tolerability and transgene expression of systemic non-viral DNA delivery

Sujata Senapati, Thais B. Bertolini, Michael A. Minnier, Mustafa N. Yazicioglu, David M. Markusic, Rui Zhang, Joan Wicks, Ali Nahvi, Roland W. Herzog, Matthew C. Walsh, Pedro J. Cejas, and Sean M. Armour

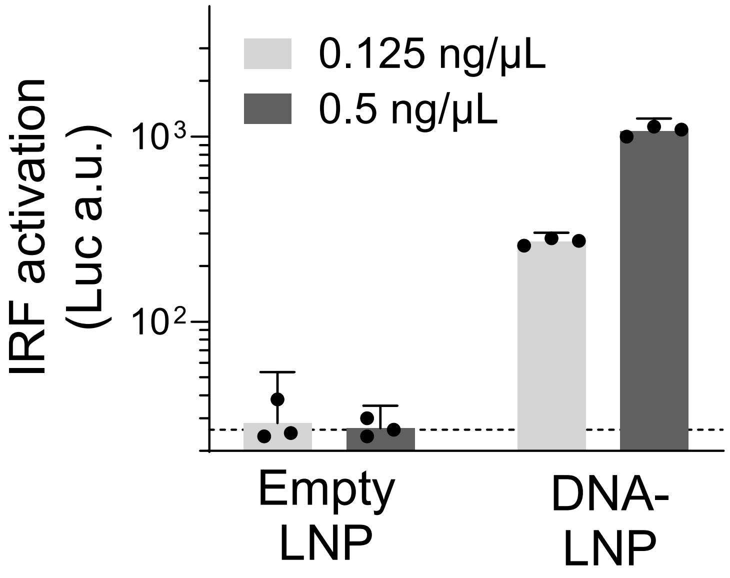

Figure S1. THP1-Dual cells were treated with either LNP encapsulating no payload (empty LNP) or plasmid DNA (DNA-LNP) at the indicated concentrations. IRF activation was measured by luminescence. Data shown as geomean with 95% CI; luminescence of untreated cells shown in dashed line.

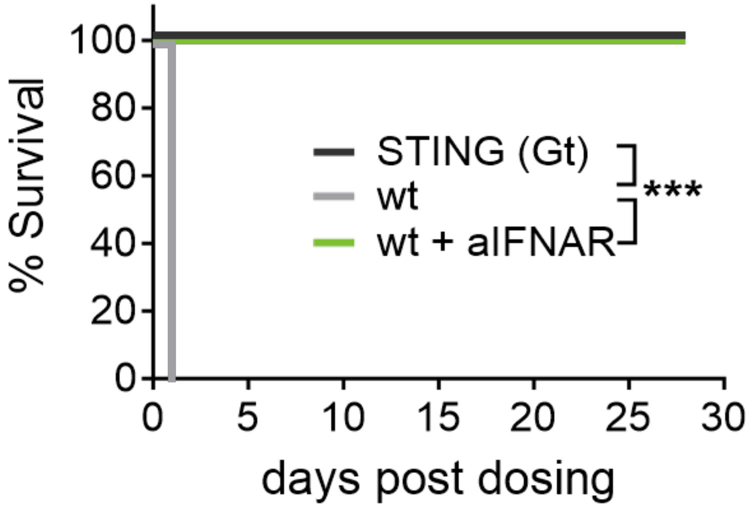

<u>Figure S2.</u> Wild-type (wt) and STING-deficient Goldenticket (Gt) mice (n=5 per group) were intravenously (tail vein) dosed with 100 µg of human Factor IX transgene encapsulated in LNP formulation (DNA-LNP). Wt mice were either untreated or treated with 300 µg per dose of anti-IFNAR intraperitoneally at 3 hours prior to DNA-LNP dosing. Survival of mice was followed out to 30 days post-dosing with of DNA-LNP. Survival data was analyzed by log-rank (Mantel-Cox) test; *** denotes $p<0.0001$
