# Supplementary material for: Inhibition of IFNAR-JAK signaling enhances tolerability and transgene expression of systemic non-viral DNA delivery
Source: Mol Ther Nucleic Acids. 2025 Mar 5;36(2):102502. doi: 10.1016/j.omtn.2025.102502 (PMC11979999; doi:10.1016/j.omtn.2025.102502)
Supplement: Document S2. Article plus supplemental information [file mmc2.pdf]

Molecular Therapy
Nucleic Acids
Original Article

Inhibition of IFNAR-JAK signaling enhances tolerability and transgene expression of systemic non-viral DNA delivery

Sujata Senapati,[1] Thais B. Bertolini,[2] Michael A. Minnier,[1] Mustafa N. Yazicioglu,[1] David M. Markusic,[2] Rui Zhang,[1] Joan Wicks,[3] Ali Nahvi,[1] Roland W. Herzog,[2] Matthew C. Walsh,[1] Pedro J. Cejas,[1] and Sean M. Armour[1]

[1]Discovery Group, Spark Therapeutics, Philadelphia, PA, USA; [2]Department of Pediatrics, Indiana University School of Medicine, Indianapolis, IN, USA; [3]Gene Therapy Research, Spark Therapeutics, Philadelphia, PA, USA

Lipid nanoparticles (LNPs) have demonstrated significant therapeutic value for non-viral delivery of mRNA and siRNA. While there is considerable interest in utilizing LNPs for delivering DNA (DNA-LNPs) to address a broad range of genetic disorders, acute inflammatory responses pose significant safety concerns and limit transgene expression below therapeutically relevant levels. However, the mechanisms and immune signaling pathways underlying DNA-LNP-triggered inflammatory responses are not well characterized. Through the use of gene-targeted mouse models, we have identified cGAS-STING and interferon-α/β receptor (IFNAR) pathways as major mediators of acute inflammation triggered by systemic delivery of DNA-LNPs. cGAS-STING activation induces expression of numerous JAK-STAT-activating cytokines, and we show that treatment of mice with the JAK inhibitors ruxolitinib or baricitinib significantly improves tolerability to systemically delivered DNA-LNPs. Furthermore, specific inhibition of IFNAR signaling enhances both DNA-LNP tolerability and transgene expression. Utilization of JAK inhibitors or IFNAR blockade represent promising strategies for enhancing the safety and efficacy of non-viral DNA delivery for gene therapy.

INTRODUCTION

Lipid nanoparticles (LNPs) have emerged as the most advanced non-viral platform[1–4] for nucleic acid delivery. This is exemplified by the US Food and Drug Administration (FDA) approval of an LNP-delivered RNAi therapeutic for hereditary transthyretin-mediated amyloidosis (Onpattro [patisiran], Alnylam Pharmaceuticals) in 2018[5] and approvals of two LNP-delivered mRNA vaccines for SARS-CoV-2 (Comirnaty, BioNTech Manufacturing GmbH and Pfizer; Spikevax, Moderna) in 2021.[6,7] Non-viral DNA delivery vehicles offer the promise of long-term gene expression to treat a broad range of genetic diseases with potential advantages over viral vectors in redosability, payload capacity, cost of production, and cell-type targeting.[8–10]

It has long been recognized that cytosolic DNA, both microbial and endogenous, can trigger innate immune responses,[11,12] and early efforts to deliver plasmid DNA using nonviral vehicles resulted in significant inflammation.[13–15] More recently, specific molecular mediators of DNA-triggered innate immune responses, including Toll-like receptor 9 (TLR9),[16] cyclic guanosine monophosphate-adenosine monophosphate synthetase (cGAS)[17] and the absent in melanoma 2 (AIM2) inflammasome[18] have been identified. Downstream mediators, including the endoplasmic reticulum membrane protein stimulator of interferon (IFN) genes (STING)/TMEM173[19] and various cytokines that signal through the Janus kinase/signal transducer and activator of transcription (JAK-STAT) signaling pathway, including interleukin-6 (IL-6), IFN-γ, and type I IFNs[20,21] have also been implicated in DNA-triggered pathology. Induction of IFNs and inflammasome-mediated gasdermin D (GSDMD) pore formation constitute significant causes of cytosolic DNA-triggered cytotoxicity through multiple programmed cell death pathways.[22–24] Because inflammatory responses pose a significant barrier to productive delivery of DNA therapeutics, it is necessary to evaluate DNA-LNP-associated inflammatory responses and characterize mechanisms driving those responses so that mitigating strategies can be designed.

Here, we have evaluated acute cytokine expression and tolerability profiles of systemically delivered DNA-LNPs in various mouse models, including those deficient in innate immune sensors. We have identified the cGAS-STING pathway as a significant obstacle to DNA-LNP tolerability and have demonstrated that tolerability and efficacy can be markedly improved by inhibiting type I IFN-JAK-STAT signaling activated downstream of cGAS-STING. These findings may enable approaches that ensure both the effectiveness and safety of DNA-LNP therapies.

Received 11 September 2024; accepted 28 February 2025;
https://doi.org/10.1016/j.omtn.2025.102502.

Correspondence: Pedro J. Cejas, Spark Therapeutics, Philadelphia, PA 19104, USA.
E-mail: pedro.cejas@sparktx.com
Correspondence: Sean M. Armour, Spark Therapeutics, Philadelphia, PA 19104, USA.
E-mail: sean.armour@sparktx.com

Published by Elsevier Inc. on behalf of The American Society of Gene and Cell Therapy.
This is an open access article under the CC BY license (http://creativecommons.org/licenses/by/4.0/).

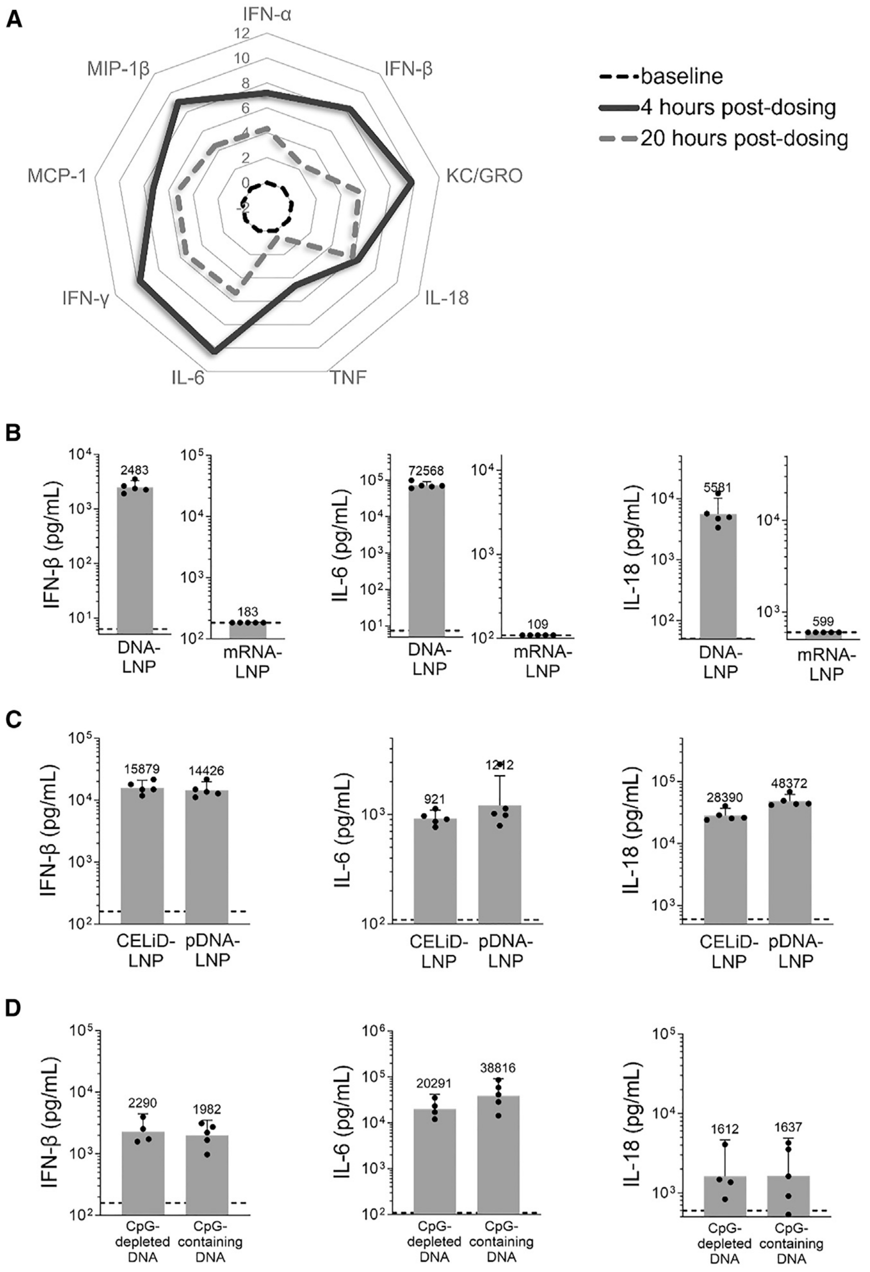

Figure 1. Systemic delivery of DNA by LNPs induces significant acute cytokine responses

C57BL/6 mice (n = 5) were intravenously (i.v., tail vein) dosed with LNP formulations encapsulating (A) 5 μg plasmid DNA (DNA-LNP); (B) 5 μg plasmid DNA or mRNA (mRNA-LNP); (C) 25 μg closed-ended linear duplex DNA (CELiD-LNP) or 25 μg plasmid DNA (DNA-LNP); and (D) 5 μg CpG-containing or CpG-depleted plasmid DNA. Cytokine and chemokine levels in plasma were measured at 3–4 h or 20 h post-dosing. Data shown as $\log_2$ fold increase over baseline levels (naive mice) in radar plot and as geometric mean with 95% CI in bar graphs; naive levels are shown as dashed line for each cytokine in bar graphs.

RESULTS

Systemic delivery of DNA by LNPs induces strong innate immune responses

To examine the effect of DNA payload on the activation of inflammatory signaling by LNPs, we incubated THP1-Dual reporter cells with LNPs encapsulating plasmid DNA (DNA-LNP) or LNPs with no nucleic acid payload (empty LNPs). While empty LNPs elicited no measurable IFN regulatory factor (IRF) activity, DNA-LNPs triggered strong IRF activity in a dose-dependent manner (Figure S1), demonstrating that the DNA payload, and not the lipid components, is driving the acute immune response in this *in vitro* system. To evaluate the innate immune response to systemic delivery of DNA-LNPs *in vivo*, we dosed mice intravenously (i.v.) with plasmid DNA encapsulated in LNPs. Serum analysis revealed robust induction of type I IFNs (IFN-α and IFN-β), pro-inflammatory cytokines (IFN-γ, IL-6, tumor necrosis factor [TNF], and IL-18) and chemokines (monocyte chemoattractant protein-1 [MCP-1], macrophage inflammatory protein-1β [MIP-1β], and keratinocyte-derived chemokine/growth-regulated oncogene α [KC/GROα]) at 4 h after DNA-LNP dosing, with somewhat reduced levels observed at 20 h (Figure 1A). This acute response is specific to the DNA payload, since no significant effect was observed with a similar LNP formulation encapsulating a 5-methoxyuridine (5moU)-modified mRNA payload (mRNA-LNP) (Figure 1B). It has been proposed that alternate DNA structures, such as closed-ended linear duplex DNA (CELiD) may significantly minimize the innate immune response elicited by DNA-LNPs.[25] However, we found that DNA-LNPs formulated with CELiD induced similar cytokine responses to those induced by DNA-LNPs formulated with plasmid DNA (Figure 1C). Unmethylated cytosine-phosphate-guanosine (CpG) motifs in the DNA payload can also contribute to the innate immune response.[26] However, mice dosed with LNPs encapsulating CpG-containing or CpG-depleted DNA plasmids showed similar levels of cytokine induction (Figure 1D), suggesting that the observed inflammatory response to DNA-LNPs is largely CpG independent.

Ablation of the cGAS-STING pathway dramatically reduces DNA-LNP-triggered pro-inflammatory response and improves tolerability

To characterize the mechanisms underlying *in vivo* immune responses to DNA-LNPs, we evaluated acute innate responses in various mouse models genetically deficient for selected immune

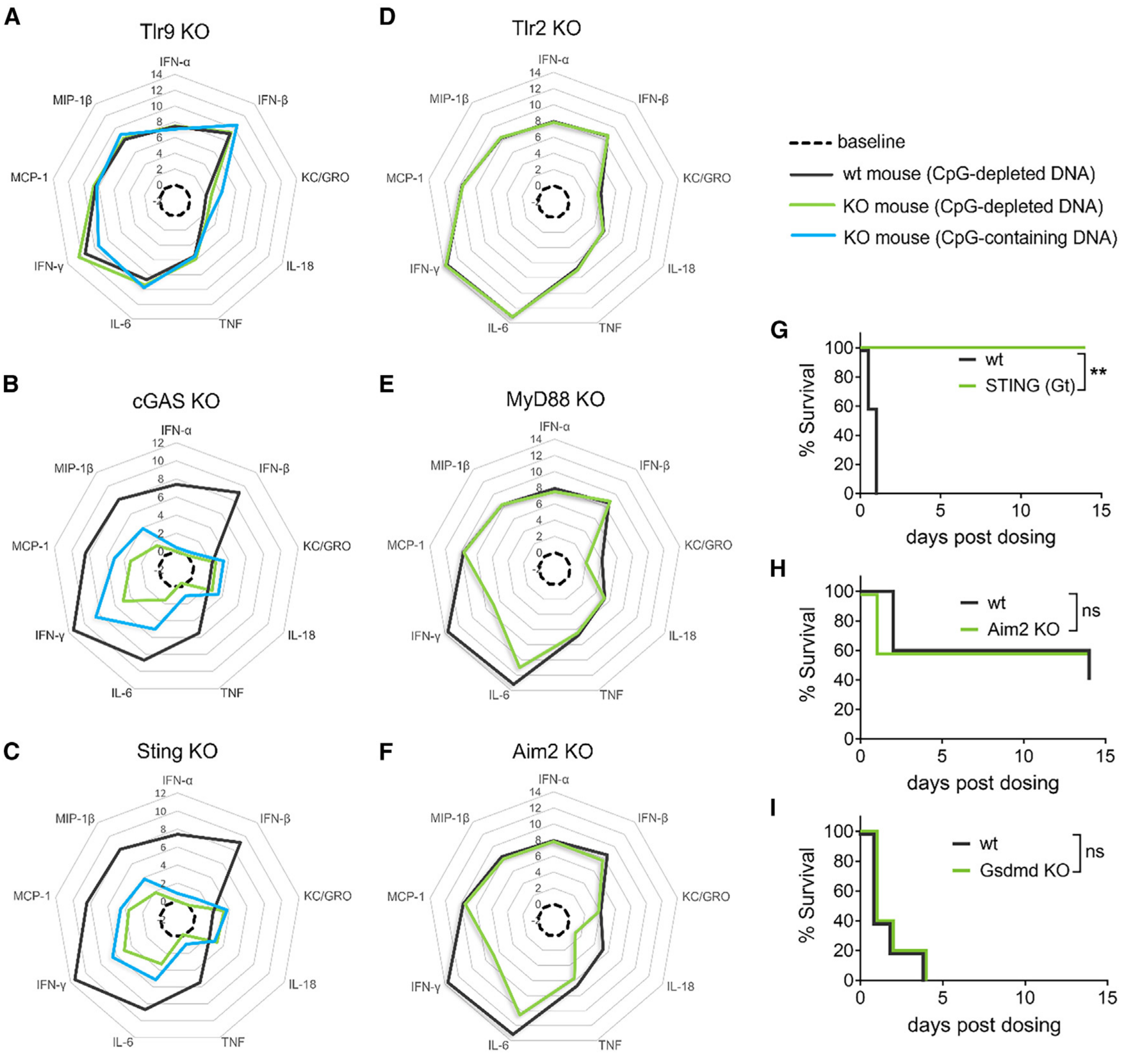

Figure 2. Ablation of the cGAS-STING, but not the TLR9, TLR2, MyD88, or inflammasome pathways, reduced pro-inflammatory cytokine levels and improved survival after systemic dosing of DNA-LNP

Wild-type (WT) and various innate-immunodeficient knockout (KO) mice (n = 5/group) were i.v. (tail vein) dosed with LNP formulation encapsulating CpG-depleted or CpG-containing hFIX transgene (DNA-LNP). (A–F) Levels of various pro-inflammatory cytokines and chemokines in WT and KO mice were measured by Luminex 4 h post-dosing with 5 μg DNA-LNP. Data represented as $\log_2$ fold increase over baseline levels (naive mice). Survival of mice in each group was followed out to 14 days post-dosing with 25 μg (H) or 50 μg (G and I) dose of DNA-LNP. Survival data were analyzed by log rank (Mantel-Cox) test; $^{**}p < 0.001$; ns, not significant.

signaling mediators. To further evaluate contributions of CpG DNA motifs, we dosed mice deficient for TLR9,[16] the endosomal receptor for unmethylated CpG DNA,[26] and found that cytokine and chemokine responses in TLR9 knockout (KO) mice were similar to those in wild-type (WT) control mice whether the plasmid DNA payload was CpG containing or CpG depleted (Figure 2A), further suggesting that innate immune responses to DNA-LNPs are CpG independent. We next evaluated mice deficient in cGAS or STING that comprise the cGAS-STING double-stranded DNA cytosolic sensor and signaling pathway. Unlike TLR9 KO mice, both cGAS KO and STING KO mice exhibited dramatic reductions in all DNA-LNP-induced cytokines (IFN-α, IFN-β, IL-6, TNF, and IFN-γ) and chemokines (MCP-1 and MIP-1β) examined, with the exceptions of KC/GROα and IL-18 (Figures 2B and 2C). Interestingly, in the absence of cGAS or STING, further diminished cytokine and chemokine levels could be observed with CpG-depleted versus CpG-containing DNA payloads, suggesting that CpG DNA motifs have minor contributions to the DNA-LNP inflammatory response (Figures 2B and 2C). Based on these results, we conducted subsequent experiments using CpG-depleted plasmid DNA as the default DNA-LNP payload. We further evaluated mice deficient for components of the TLR2-MyD88 pathway, which has been implicated in LNP-mediated inflammatory responses.[27] While TLR2 KO mice showed no defect in DNA-LNP-induced inflammatory responses (Figure 2D), MyD88 KO mice

exhibited minor reductions in DNA-LNP-induced KC/GROα and IL-6 and more substantial reduction in IFN-γ induction compared to levels in WT control mice (Figure 2E). Significant induction of IL-18 secretion after DNA-LNP dosing may be responsible for driving IFN-γ[28] expression and is indicative of inflammasome activation.[29] Therefore, we evaluated mice deficient for the DNA-sensing inflammasome AIM2[18] and found that they exhibited a minor reduction in DNA-LNP-induced IL-6 and more substantial reductions in both IFN-γ and IL-18 compared to levels in WT control mice (Figure 2F).

To determine the contribution of the cGAS-STING pathway to tolerability to higher doses of DNA-LNP, we systemically dosed WT control mice and STING *Goldenticket* (Gt) null mutant mice[30] with 50 μg DNA-LNP. While all WT control mice succumbed within 1 day, all STING(Gt) mice survived to the 14-day endpoint (Figure 2G). In another study, to evaluate the role of the AIM2 inflammasome in DNA-LNP tolerability, we dosed WT control mice and AIM2-deficient mice with 50 μg DNA-LNP but found no difference in susceptibility (Figure 2H). Considering alternative inflammasomes may be participating in the response to DNA-LNPs, we employed mice deficient in Gsdmd, the essential downstream mediator of multiple inflammasomes, including AIM2.[31] However, like AIM2-deficient mice, Gsdmd KO mice showed reduced IL-18 and IFN-γ levels (data not shown) and no difference in susceptibility compared to WT control mice (Figure 2I). Therefore, while inflammasomes likely contribute to the response to DNA-LNP, it appears that activation of the cGAS-STING pathway is the major barrier to tolerability.

Inhibiting JAK improves DNA-LNP tolerability

There are currently no approved therapeutics that directly target cGAS-STING activation. However, since we found that DNA-LNP-induced cGAS-STING activation leads to the induction of multiple inflammatory cytokines that signal through the JAK-STAT pathway, we employed FDA-approved JAK inhibitors (JAKi) ruxolitinib[32,33] and baricitinib[34,35] to determine whether JAK-STAT cytokine receptor signaling is a barrier to DNA-LNP tolerability. We dosed WT control mice systemically with 50 μg DNA-LNP with or without ruxolitinib or baricitinib JAKi treatment. We similarly treated STING(Gt) mice with the same dose of DNA-LNP. We found that while JAKi did not have significant effects on cytokine induction other than minor reduction in IFN-γ (Figures 3A and 3B), the mice treated with JAKi, like STING(Gt) mice, exhibited 100% survival, while WT control mice showed only 20% survival (Figure 3C). The results indicate that blocking JAK signaling downstream of the cGAS-STING pathway improves tolerability to DNA-LNPs. Importantly, JAKi-treated and STING(Gt) mice showed similar plasma human factor IX (hFIX) levels at 2 and 4 weeks post-DNA-LNP dosing (Figure 3D), indicating that JAKi treatment recapitulates the effect of STING deficiency on DNA-LNP tolerability without any change in efficacy.

Targeting the type I IFN pathway improves DNA-LNP tolerability and transgene expression

We next aimed to identify the contributions of JAK/STAT-signaling cytokines IL-6, IFN-γ, and IFN-α/β to the lethal inflammatory responses observed following systemic DNA-LNP dosing. IL-6 is both strongly induced in response to systemic DNA-LNP and has an established role in acute inflammatory pathology.[36] However, when we dosed IL-6-deficient (IL-6 KO) mice with 50 μg DNA-LNP, we found, surprisingly, that survival was not improved compared to WT control mice (Figure 4A). IFN-γ KO mice likewise exhibited no improvement in survival compared to WT control mice, while IFN-α/β receptor (IFNAR) KO mice showed complete rescue, with all animals surviving to the 30-day endpoint (compared to only 40% of WT control mice) (Figure 4B).

To determine whether pharmacological inhibition of IFNAR could replicate the increased tolerability observed in IFNAR KO mice, we administered two escalating doses of hFIX-expressing DNA-LNP in C57BL/6 mice treated with anti-IFNAR blocking antibody. Anti-IFNAR treatment resulted in acutely reduced plasma IFN-γ levels 3 h post-DNA-LNP dosing, but the induction of other cytokines and chemokines measured was unaffected (Figure 4C). While 80% of untreated mice and 100% of anti-IFNAR-treated mice tolerated the lower 25 μg DNA-LNP dosing, only 20% of untreated mice (compared with 100% of treated mice) survived to the endpoint after the higher dose of DNA-LNP (Figure 4D). The protective effect of anti-IFNAR treatment was observed even at a DNA-LNP dose as high as 100 μg (Figure S2). We assessed the effect of anti-IFNAR pre-treatment on the efficacy of DNA-LNP and observed a significant boost in plasma hFIX transgene expression compared to no pre-treatment across all time points out to 6 weeks post-DNA-LNP dosing (Figure 4E). Importantly, the JAKi baricitinib alone did not have a positive effect on hFIX expression levels but did not affect the boost in transgene expression observed with anti-IFNAR treatment alone (Figure 4F). Additionally, the boosting effect of anti-IFNAR could be generalized for DNA delivery by LNPs, as it was observed with transgenes other than hFIX (Figure 4G), different mouse strains, and other lipid chemistries (Figure 4H). In contrast, anti-IFNAR did not improve efficacy in mice that received adeno-associated virus (AAV)-mediated gene delivery (Figure 4I). These results indicate that employing antibody-mediated IFNAR blockade not only improves DNA-LNP tolerability but, in contrast to JAKis, enhances DNA-LNP transgene expression.

DISCUSSION

The innate inflammatory response triggered by systemic DNA-LNP delivery is a major hurdle to advancing application of LNPs to DNA-based gene therapy. High cytokine and chemokine levels in the plasma following systemic dosing of DNA-LNPs led us to hypothesize that activation of innate immune sensors upon endosomal or cytosolic release of DNA payloads may limit DNA-LNP tolerability and efficacy. Evaluation of immune responses in multiple KO mouse models identified the cGAS-STING pathway as a major barrier to tolerability of DNA-LNPs, while the TLR9-MyD88 and the AIM2 inflammasome pathways contribute to the innate immune response but do not appear to significantly limit tolerability. MyD88 functions downstream of many TLR and IL-1R superfamily members.[37] The observed reductions in IL-6 and KC in MyD88 KO

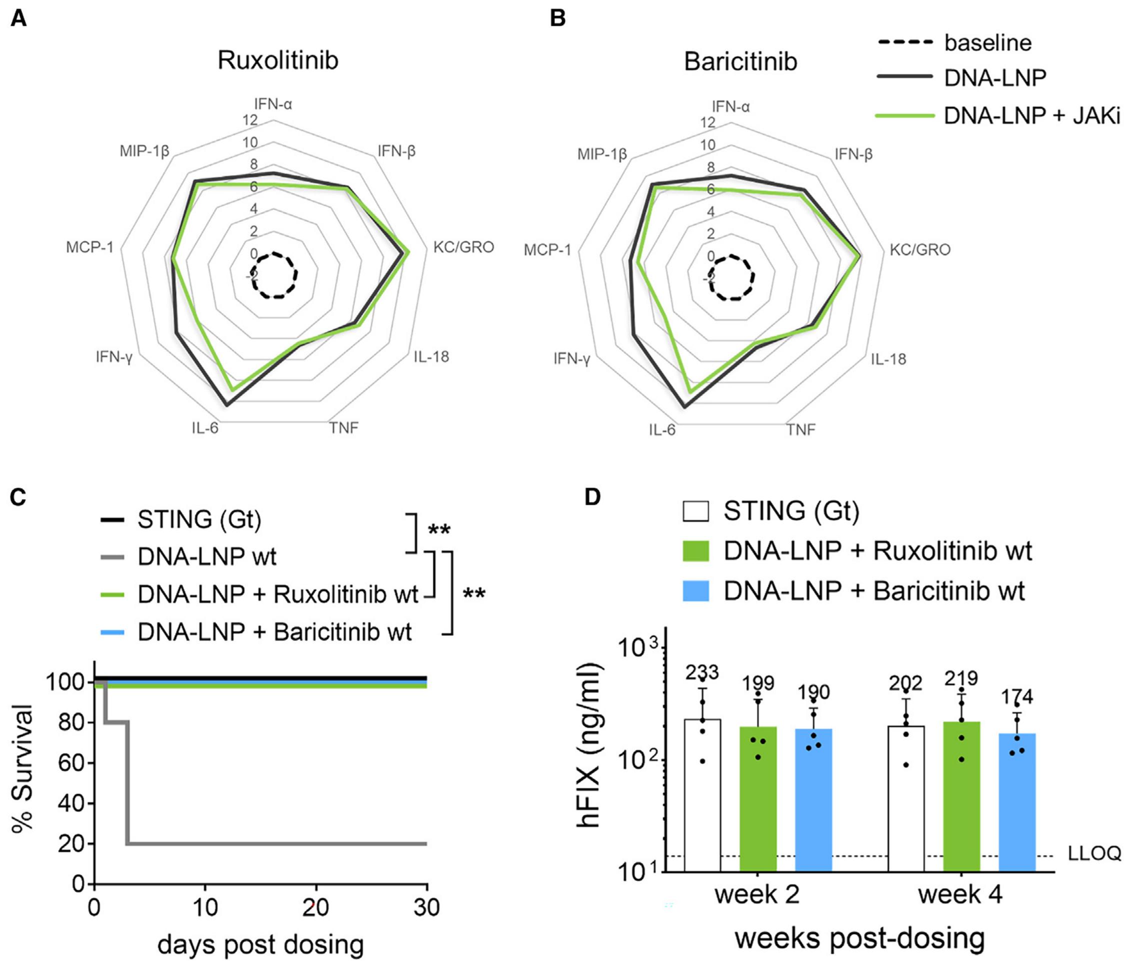

Figure 3. Targeting Janus kinase improves tolerability of DNA-LNP

WT and STING-deficient *Goldenticket* (STING(Gt)) mice (n = 5/group) were i.v. dosed via tail vein with 50 μg hFIX transgene encapsulated in LNP. WT mice were either untreated or treated orally with 200 μg/dose of Janus kinase (JAK) inhibitors (ruxolitinib or baricitinib) four times (30 min prior to and days 1, 2, and 3 post-DNA-LNP dosing). Plasma cytokine levels were measured at 4 h post-dosing in WT mice treated with either (A) ruxolitinib or (B) baricitinib. Data represented as $\log_2$ fold increase over baseline levels (naive mice). (C) Survival of mice in each group was followed out to 30 days. (D) hFIX protein level in blood plasma of all groups was measured by ELISA at weeks 2 and 4 post-dosing. Survival data were analyzed by log rank (Mantel-Cox) test, and hFIX data was analyzed using ordinary one-way ANOVA with Tukey's post hoc tests for multiple comparisons at each time point; $^{**}p < 0.001$; ns, not significant.

mice treated with DNA-LNP suggest there might be other pathways[38] that make minor contributions to the DNA-LNP-driven immune response. IFN-γ production is stimulated by cytokines (e.g., IL-18, IFN-α/β) secreted by immune cells and requires contributions from multiple upstream signaling pathways.[39] As such, it is not surprising that the absence of AIM2 or MyD88 results in a significant reduction in IFN-γ levels. It has been suggested that TLR9-MyD88 activation by unmethylated CpG sequences might significantly contribute to immune activation against viral vectors like AAV.[40] However, our findings indicate that in the context of DNA-LNPs, TLR9 plays only a minor role in the innate immune response, and that this role is observed exclusively in the absence of the cGAS-STING pathway, which is the primary driver of inflammation induced by DNA-LNPs.

It is possible that multiple cytokines interact to drive JAK-STAT signaling in a manner that limits tolerability. Therefore, we explored JAK-STAT signaling inhibition (JAKi) as a potential intervention to enhance DNA-LNP tolerability. Our findings revealed that while JAKi had little significant impact on the cytokine response triggered by cytosolic DNA and the cGAS-STING pathway, it did prevent mortality, presumably by disrupting positive cytokine feedback loops associated with high-dose DNA-LNP. The expression of type I IFNs is also highly induced by DNA-LNPs and genetic ablation of type I IFNAR is sufficient to improve tolerability. While anti-IFNAR treatment prior to DNA-LNP dosing did not significantly affect acute cytokine induction, likely because it acts downstream of cGAS-STING cytokine induction, anti-IFNAR blocking antibody improved survival. These results suggested that type I IFNs significantly

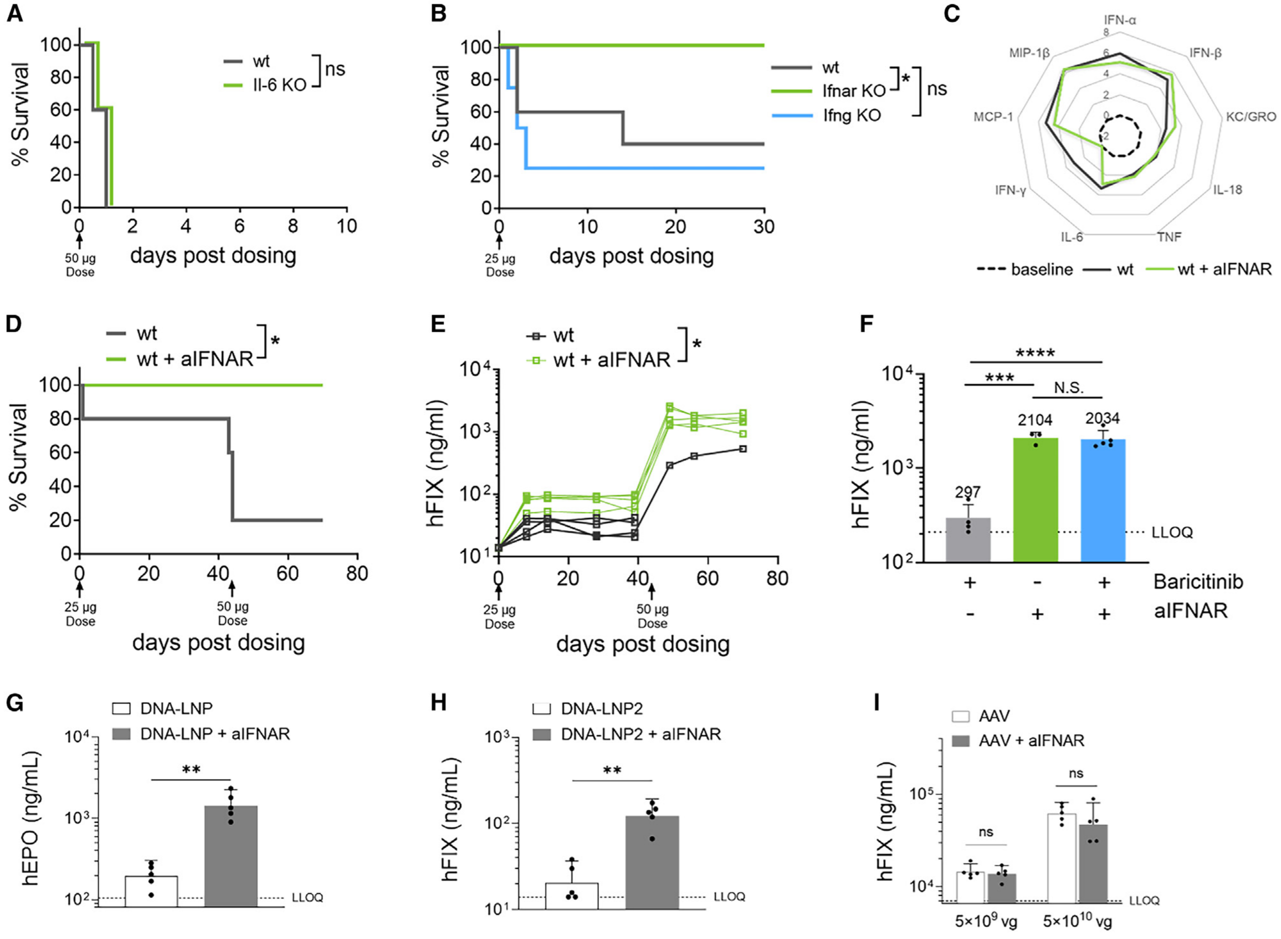

Figure 4. Targeting the type I interferon pathway improves DNA-LNP tolerability and transgene expression

WT, IL-6 KO, IFNAR KO and IFN-γ KO mice (n = 5/group) were i.v. dosed via tail vein with LNP formulation encapsulating 25–50 μg hFIX transgene (DNA-LNP). (A and B) Survival for each group of mice was followed out to 10–30 days post-dosing and analyzed by log rank (Mantel-Cox) test; $^{*}p < 0.01$; ns, not significant. (C–E) WT mice were either untreated or treated once intraperitoneally (i.p.) with 300 μg/dose of anti-mouse IFNAR antibody (aIFNAR) 3 h prior to dosing with 25 μg DNA-LNP on day 0. (C) Plasma cytokine levels measured at 4 h post-dosing are represented as log2 fold increase over baseline levels in naive mice. (D and E) WT mice were redosed with 50 μg DNA-LNP at day 41 and (D) survival was followed out to 70 days post-dosing and analyzed by log rank (Mantel-Cox) test; $^{*}p < 0.01$; ns, not significant. (E) hFIX levels in blood plasma were measured by ELISA at the indicated time points for all surviving mice and analyzed using ordinary one-way ANOVA with Tukey's post hoc tests for multiple comparisons at all time points, with $^{*}p < 0.01$. (F) WT mice were treated with 300 μg/dose of anti-IFNAR (i.p. at 3 h prior to DNA-LNP dosing), 200 μg/dose of baricitinib (orally at 90 min prior to dosing and daily from days 1–6 post-dosing with 50 μg DNA-LNP) or both. hFIX levels in blood plasma of surviving mice were measured by ELISA after 7 days and analyzed using ordinary one-way ANOVA with Tukey's post hoc tests, with $^{***}p < 0.001$; $^{****}p < 0.00001$; ns, not significant. (G) WT BALB/c background mice (n = 5/group) were i.v. dosed with 50 μg human erythropoietin (hEPO)-expressing plasmid DNA encapsulated in DNA-LNP and plasma hEPO levels measured in plasma by ELISA at 7 days post-dosing. (H) WT mice (n = 5/group) were i.v. dosed with hFIX-expressing plasmid DNA encapsulated in LNP2 (DNA-LNP2), and (I) WT mice (n = 5/group) were dosed with 5×10^{10} vector genome copies of hFIX-expressing AAV. hFIX levels were measured in plasma by ELISA at 7 days post-dosing. All animals were either untreated or treated with 300 μg/dose of anti-IFNAR i.p. at 3 h prior to DNA-LNP dosing. Data are represented as geometric means and 95% CIs and were analyzed using Mann-Whitney t tests, with $^{**}p < 0.05$; ns, not significant.

contribute to acute DNA-LNP-triggered mortality. However, excessive IFNAR signaling alone may not be sufficient to induce mortality and may require interaction with other factors induced by DNA-LNPs. The precise cause of mortality in mice treated with high-dose DNA-LNP remains unclear. Our preliminary analyses of these animals show extensive coagulation necrosis in hepatocytes and lipid vacuolation in liver tissue (data not shown). Coagulation pathways have been reported to be imbalanced in pathophysiological states and can lead to disseminated intravascular coagulation (DIC), resulting in diffuse bleeding and sepsis.[41] Initiation of DIC has been linked to STING activation and type I IFNs, potentially identifying the mechanism underlying the mortality with high doses of DNA-LNP.[42,43]

DNA-LNP transgene expression efficacy was significantly enhanced with anti-IFNAR treatment, and enhanced expression was surprisingly

durable, persisting for weeks after the acute IFN response. The mechanism behind this improvement in efficacy remains unclear and requires further investigation. While it is well known that type I IFNs exert antiviral effects by globally suppressing translation through the expression of IFN-stimulated gene (ISG)-encoded proteins,[44] this is unlikely to be the mechanism since the higher transgene expression is maintained at much later time points, when type I IFNs and ISGs are no longer upregulated. Type I IFNs also mediate the expression of apoptosis-inducing proteins, leading to cell death.[45,46] Therefore, blockade of IFNAR-mediated cytotoxicity may contribute to enhanced DNA-LNP transgene expression. Blockade of IFNAR has been reported to prevent $CD8^+$ T cell activation following AAV-mediated gene delivery[47]; however, since AAV does not induce a strong acute IFN response in mice,[47] it is not surprising that anti-IFNAR did not enhance the expression of AAV-delivered transgene. It is possible that IFNAR blockade could enhance efficacy for viral vectors that are strong IFN inducers, such as lentiviruses,[48] or for AAV at higher doses and in contexts where adaptive responses limit efficacy.[49]

It is also unclear why blocking JAK activity with the JAKi ruxolitinib or baricitinib did not result in a similar enhancement of transgene expression. One possibility is that the pharmacodynamics of these small molecules were insufficient to provide the necessary level of inhibition of JAK signaling downstream of IFNAR to enhance expression. Alternatively, it is possible that multiple JAK-STAT cytokine pathways interact to both positively and negatively affect transgene expression and that broad JAK inhibition results in no net effect. However, our finding that baricitinib treatment does not counteract the positive effect of anti-IFNAR would tend not to support a significant role for this effect. Additional ongoing work aims to better understand this mechanism(s) underlying these observations.

Application of DNA-LNP to gene therapy will require prevention or suppression of the innate immune response triggered by DNA payloads. Attempts have been made to limit the intrinsic inflammatory nature of DNA, such as employing CELiD, which we found induces inflammatory cytokines at levels similar to those of standard plasmid DNA. This result may not be surprising since CELiD DNA is double stranded. More recently, it has been reported that engineered single-stranded DNA is significantly better tolerated than plasmid DNA when employed as a payload for DNA-LNPs[50]. Given that even single-stranded DNAs may form secondary structures capable of activating DNA sensors, it remains unclear whether modifying the format of DNA in this manner will sufficiently limit cGAS-STING activation such that additional prophylactic intervention is not required. In the absence of effective direct inhibitors of the cGAS-STING pathway, alternative therapeutic strategies, such as blocking JAK or IFNAR signaling, may be viable solutions. Various JAKis (e.g., ruxolitinib, baricitinib, tofacitinib, abrocitinib[51]) and at least one anti-IFNAR drug (anifrolumab)[52] are currently approved for various inflammatory indications unrelated to gene therapy but could be incorporated into pre-treatment regimens to support DNA-LNP therapeutics. Our findings using anti-IFNAR treatment demonstrate that effective suppression of the innate immune response to DNA-LNPs is not only critical for tolerability but also may be a key element to achieve therapeutic levels of transgene expression.

MATERIALS AND METHODS

LNP formulation and characterization

LNPs were prepared using model ionizable lipids, branched CKK-E12 (unless otherwise stated) dioleoylphosphatidylethanolamine, cholesterol, and C14-polyethylene glycol (PEG) (Avanti Polar Lipids, Alabaster, AL) at a molar ratio of 35:16:50:2.4. C18-PEG-GalNAc (Sussex Research, Ottawa, Canada) was incorporated into the LNP for some studies. The lipids were dissolved in ethanol and combined with 50 mM, pH 3 citrate buffer containing nucleic acid payload at a ratio of 3:1 (aqueous:ethanol) using a microfluidic device (Precision Nanosystems, Vancouver, Canada). The payload used was either 5moU modified erythropoietin (EPO) mRNA payload (TriLink BioTechnologies, San Diego, CA), plasmid DNA (codon optimized hFIX or factor VIII nanoplasmid under the control of apolipoprotein E (ApoE) enhancer and human α1 antitrypsin (haaT) promoter, with or without CpG), human EPO (Aldevron, Fargo, ND), or human factor VIII CELiD (SQY Therapeutics, Montigny-Le-Bretonneux, France). After synthesis, formulations were purified in dialysis cassettes (Slide-A-Lyzer, 20 KDa molecular weight cutoff [MWCO], Thermo Fisher Scientific, Waltham, MA) with phosphate-buffered saline (PBS) (pH 7.4, no Ca^{2+} or Mg^{2+}) overnight for the removal of excess ethanol. Formulations were then concentrated using Amicon filters (20 kDa MWCO; Sigma-Aldrich, St Louis, MO). We added 10% sucrose (prepared from powdered sucrose stock; Sigma-Aldrich) to the formulations prior to storage at −80°C.

All formulations were characterized for particle size and polydispersity index (PDI) using Zetasizer (Nano-ZS, Malvern Instruments, Malvern, UK) and encapsulation efficiency using Ribogreen assay (Thermo Fisher Scientific). Briefly, Ribogreen reagent was added to LNP samples in a 96-well plate following treatment with 2% Triton X-100 and Tris-EDTA (TE) buffer in separate reaction wells. The plate was measured for fluorescence. Nucleic acid concentrations in each well were interpolated on a standard curve. Encapsulated efficiency was calculated using the concentration values for the Triton X-100 and TE wells for each sample. DNA preps and final DNA-LNP formulations were determined to be below detection limits for endotoxin by Endosafe nexgen-PTS (Charles River Laboratories, Wilmington, MA) assay. The average size of all LNPs used in this study was approximately 60 nm and the PDI was 0.17. The encapsulation efficiency was 85% or higher.

In vitro assay

THP1-Dual cells (thpd-nfis, InvivoGen, San Diego, CA) were plated at a density of 80,000 cells per well in a U-bottom 96-well plate (Falcon, 351177). The cells were treated with LNP formulations at the desired concentration and 1 μg/mL recombinant ApoE4 (350-04, Peprotech, Cranbury, NJ). IRF activity was measured by reading luminescence using QUANTI-Luc (rep-qlc4lg1, Invitrogen) according to the manufacturer's instructions.

Animals

C57BL/6 or Balb/c mice and various innate-immune KO mouse strains (strain nos. 026554, 025805, 013144, 004650, and 009088) were purchased from The Jackson Laboratory (Bar Harbor, ME). The KO mice were age and gender matched with the WT strain for all studies. Animals were fed *ad libitum* and were housed in solid bed cages in rooms under controlled environmental conditions. All research conducted was in accordance with the Institutional Animal Care and Use Committee guidelines at Indiana University and the Guide for the Care and Use of Laboratory Animals (National Research Council) at Spark Therapeutics. For survival studies, mice who lost more than 20% of their original body weight or were under severe distress (cold to touch, severely hunched, lethargic, having difficulty breathing) were euthanized.

Treatment administration

LNP formulations diluted to specific concentrations were injected i.v. (200–260 μL/mouse) into mice via the tail vein using 29 g, 3/10 cm^3 insulin syringes (BD Biosciences, Franklin Lakes, NJ) after gentle warming of the animals using a heat lamp for 3 min. AAV-Spark 100 vector expressing hFIX under the control of ApoE-hAAT was also dosed i.v into mice via the tail vein. Various pre-treatments baricitinib (MedChemExpress, Monmouth Junction, NJ), ruxolitinib (MedChemExpress), anti-mouse IFNAR (clone MAR1-5A3, Bio-XCell, Lebanon, NH) was injected oral or intraperitoneally (i.p.) at the indicated dose and volume. Baricitinib was dissolved in a solution of 30% PEG 400, 0.5% Tween 80, and 5% polypropylene glycol. Ruxolitinib was dissolved in PBS. Dexamethasone (200 μg/dose) dissolved in PBS was used as pre-treatment (i.p.) 1 h prior to dosing with LNP in all studies.

Blood collection

Blood was collected from mice using the submandibular vein into lithium heparin tubes. The tubes were then centrifuged at 9,800 × *g* for 10 min at 4°C, and the plasma was collected to be stored at −80°C until further analysis.

Luminex assay for cytokine measurement

A Luminex assay was performed according to the protocol in the kit insert (ProcartaPlex Mouse and Rat Mix & Match, MAN0025393, Thermo Fisher Scientific).

hFIX ELISA

hFIX plasma concentration was measured using a sandwich ELISA. Polyclonal anti-hFIX antibody (FIX-EIA-C, Affinity Biologicals, Ancaster, Canada) was diluted in 0.05 M carbonate-bicarbonate buffer (C3041, Sigma-Aldrich) and added to each well of a 96-well enzyme immunoassay (EIA)/radioimmunoassay plate (3590, Corning, Corning, NY). The plate was incubated at room temperature (RT) for 2 h. Human pooled normal plasma (0010-5, George King Bio-Medical, Overland Park, KS) was diluted in pooled control mouse plasma (MSE02PLLH-0102342, BioIVT, Westbury, NY) to create a 2-fold, 7-point standard curve. Standard curve preparations and mouse plasma samples were then diluted in sample diluent (HEPES, NaCl, NaEDTA, and BSA in water). The ELISA plate was washed using a microplate washer (405LS, Biotek, Winooski, VT). Diluted standard and sample were transferred to the plate and incubated at RT for 90 min. Following this incubation, the plate was washed as described above. Polyclonal anti-hFIX antibody (FIX-EIA-D, Affinity Biologicals) was diluted in sample diluent, transferred to each well of the ELISA plate, and incubated at RT for 90 min. The plate was washed as described above. A 1-Step Ultra TMB (34029, Thermo Fisher Scientific) was added to each well of the ELISA plate and was incubated at RT in the dark for 7 min We added 1 M sulfuric acid (S25897, Thermo Fisher Scientific) to each well of the ELISA plate. The ELISA plate was measured for absorbance at 450 nm using a plate reader (Synergy H1, Biotek). A four-parameter standard curve was generated using the plate reader software. The hFIX concentration in each sample was interpolated using the standard curve.

hEPO ELISA

The hEPO concentration in plasma was measured following the standard manufacturer's protocol (DEP00, R&D Systems, Minneapolis, MN).

Statistical analysis

hFIX transgene data presented as geometric means on a logarithmic scale were log transformed and compared using one-way ANOVA with Tukey's post hoc tests for multiple comparisons. The survival data were analyzed by log rank (Mantel-Cox) tests.

DATA AVAILABILITY

The raw data supporting the conclusions of this article will be made available by the authors upon request.

ACKNOWLEDGMENTS

This research was funded by Spark Therapeutics. The authors acknowledge the support from the Non-clinical Research Operations, Bioanalytical Sciences, and Medical Communications teams at Spark Therapeutics.

AUTHOR CONTRIBUTIONS

Conceptualization: R.Z., A.N., R.W.H., M.C.W., and P.J.C.; design, synthesis, and formulation of study materials: M.A.M., M.N.Y., R.Z., and M.C.W.; *in vivo* study design and direction: S.S., T.B.B., D.M.M., and M.C.W.; data acquisition and analysis: S.S., T.B.B., M.A.M., J.W., and M.C.W.; writing, review, & editing: S.S., M.C.W., R.W.H., and P.J.C.; resources and supervision: S.M.A.

DECLARATION OF INTERESTS

S.S., M.A.M., M.N.Y., D.M.M., J.W., M.C.W., P.J.C., and S.M.A. are employed or were employed at the time the studies were conducted by Spark Therapeutics, a member of the Roche group, and may own stocks/options in the company. R.W.H. serves as the editor-in-chief of *Molecular Therapy*. R.Z., M.C.W., P.J.C., and S.M.A. hold a patent on IFNAR's enhancement of non-viral DNA tolerability and expression.

SUPPLEMENTAL INFORMATION

Supplemental information can be found online at https://doi.org/10.1016/j.omtn.2025.102502.

REFERENCES

1. Pozzi, D., and Caracciolo, G. (2023). Looking Back, Moving Forward: Lipid Nanoparticles as a Promising Frontier in Gene Delivery. ACS Pharmacol. Transl. Sci. 6, 1561–1573. https://doi.org/10.1021/acsptsci.3c00185.

2. Wang, C., Pan, C., Yong, H., Wang, F., Bo, T., Zhao, Y., Ma, B., He, W., and Li, M. (2023). Emerging non-viral vectors for gene delivery. J. Nanobiotechnol. *21*, 272. https://doi.org/10.1186/s12951-023-02044-5.

3. Yin, H., Kanasty, R.L., Eltoukhy, A.A., Vegas, A.J., Dorkin, J.R., and Anderson, D.G. (2014). Non-viral vectors for gene-based therapy. Nat. Rev. Genet. *15*, 541–555. https://doi.org/10.1038/nrg3763.

4. Tenchov, R., Bird, R., Curtze, A.E., and Zhou, Q. (2021). Lipid Nanoparticles from Liposomes to mRNA Vaccine Delivery, a Landscape of Research Diversity and Advancement. ACS Nano *15*, 16982–17015. https://doi.org/10.1021/acsnano.1c04996.

5. Akinc, A., Maier, M.A., Manoharan, M., Fitzgerald, K., Jayaraman, M., Barros, S., Ansell, S., Du, X., Hope, M.J., Madden, T.D., et al. (2019). The Onpattro story and the clinical translation of nanomedicines containing nucleic acid-based drugs. Nat. Nanotechnol. *14*, 1084–1087. https://doi.org/10.1038/s41565-019-0591-y.

6. Parums, D.V. (2021). Editorial: First Full Regulatory Approval of a COVID-19 Vaccine, the BNT162b2 Pfizer-BioNTech Vaccine, and the Real-World Implications for Public Health Policy. Medical Science Monitor *27*. Med. Sci. Monit. *27*, e934625. https://doi.org/10.12659/MSM.934625.

7. Ledford, H. (2020). Moderna COVID vaccine becomes second to get US authorization. Nature. Epub ahead of print. https://doi.org/10.1038/d41586-020-03593-7.

8. Dilliard, S.A., and Siegwart, D.J. (2023). Passive, active and endogenous organ-targeted lipid and polymer nanoparticles for delivery of genetic drugs. Nat. Rev. Mater. *8*, 282–300. https://doi.org/10.1038/s41578-022-00529-7.

9. Kulkarni, J.A., Cullis, P.R., and Van Der Meel, R. (2018). Lipid Nanoparticles Enabling Gene Therapies: From Concepts to Clinical Utility. Nucleic Acid Therapeut. *28*, 146–157. https://doi.org/10.1089/nat.2018.0721.

10. Sharma, J., and Paschalis, E.I. (2022). The Future of Non-viral Gene Delivery for the Treatment of Inherited Retinal Diseases. Mol Ther Nucleic Acids *30*, 354. https://doi.org/10.1016/j.omtn.2022.10.011.

11. Wu, J., and Chen, Z.J. (2014). Innate Immune Sensing and Signaling of Cytosolic Nucleic Acids. Annu. Rev. Immunol. *32*, 461–488. https://doi.org/10.1146/annurev-immunol-032713-120156.

12. Briard, B., Place, D.E., and Kanneganti, T.D. (2020). DNA Sensing in the Innate Immune Response. Preprint at American Physiological Society. Physiology *35*, 112–124. https://doi.org/10.1152/physiol.00022.2019.

13. Nasrullah, M., Meenakshi Sundaram, D.N., Claerhout, J., Ha, K., Demirkaya, E., and Uludag, H. (2023). Nanoparticles and Cytokine Response. Front Bioeng Biotechnol. *28*, 1243651. https://doi.org/10.3389/fbioe.2023.1243651.

14. Sakurai, F., Terada, T., Yasuda, K., Yamashita, F., Takakura, Y., and Hashida, M. (2002). The role of tissue macrophages in the induction of proinflammatory cytokine production following intravenous injection of lipoplexes. Gene Ther. *9*, 1120–1126. https://doi.org/10.1038/sj.gt.3301784.

15. Yew, N.S., Wang, K.X., Przybylska, M., Bagley, R.G., Stedman, M., Marshall, J., Scheule, R.K., and Cheng, S.H. (1999). Contribution of Plasmid DNA to Inflammation in the Lung after Administration of Cationic Lipid:pDNA Complexes. Hum. Gene Ther. *10*, 223–234. https://doi.org/10.1089/10430349950019011.

16. Hemmi, H., Takeuchi, O., Kawai, T., Kaisho, T., Sato, S., Sanjo, H., Matsumoto, M., Hoshino, K., Wagner, H., Takeda, K., and Akira, S. (2000). A Toll-like receptor recognizes bacterial DNA. Nature *408*, 740–745. https://doi.org/10.1038/35047123.

17. Ablasser, A., and Chen, Z.J. (2019). CGAS in action: Expanding roles in immunity and inflammation. Science *363*, eaat8657. https://doi.org/10.1126/science.aat8657.

18. Rathinam, V.A.K., Jiang, Z., Waggoner, S.N., Sharma, S., Cole, L.E., Waggoner, L., Vanaja, S.K., Monks, B.G., Ganesan, S., Latz, E., et al. (2010). The AIM2 inflammasome is essential for host defense against cytosolic bacteria and DNA viruses. Nat. Immunol. *11*, 395–402. https://doi.org/10.1038/ni.1864.

19. Ishikawa, H., and Barber, G.N. (2008). STING is an endoplasmic reticulum adaptor that facilitates innate immune signalling. Nature *455*, 674–678. https://doi.org/10.1038/nature07317.

20. Hu, X., li, J., Fu, M., Zhao, X., and Wang, W. (2021). The JAK/STAT signaling pathway: from bench to clinic. Signal Transduct Target Ther. *6*, 402. https://doi.org/10.1038/s41392-021-00791-1.

21. Hu, X., Zhang, H., Zhang, Q., Yao, X., Ni, W., and Zhou, K. (2022). Emerging role of STING signalling in CNS injury: inflammation, autophagy, necroptosis, ferroptosis and pyroptosis. J. Neuroinflammation *19*, 242. https://doi.org/10.1186/s12974-022-02602-y.

22. Larkin, B., Ilyukha, V., Sorokin, M., Buzdin, A., Vannier, E., and Poltorak, A. (2017). Cutting Edge: Activation of STING in T Cells Induces Type I IFN Responses and Cell Death. J. Immunol. *199*, 397–402. https://doi.org/10.4049/jimmunol.1601999.

23. Banerjee, I., Behl, B., Mendonca, M., Shrivastava, G., Russo, A.J., Menoret, A., Ghosh, A., Vella, A.T., Vanaja, S.K., Sarkar, S.N., et al. (2018). Gasdermin D Restrains Type I Interferon Response to Cytosolic DNA by Disrupting Ionic Homeostasis. Immunity *49*, 413–426.e5. https://doi.org/10.1016/j.immuni.2018.07.006.

24. Liu, J., Zhou, J., Luan, Y., Li, X., Meng, X., Liao, W., Tang, J., and Wang, Z. (2024). cGAS-STING, Inflammasomes and Pyroptosis: An Overview of Crosstalk Mechanism of Activation and Regulation. Cell Commun Signal. *22*, 22. https://doi.org/10.1186/s12964-023-01466-w.

25. Kerr, D.A., Samayoa, P., Kotin, R.M., Stanton, M.G., Alkan, O., Chiocco, M., and Rejendran, R. (2020). WO2020154645A1. https://patents.google.com/patent/WO2020154645A1/en.

26. Lipford, G.B., Heeg, K., and Wagner, H. (1998). Bacterial DNA as immune cell activator. Trends Microbiol. *6*, 496–500. https://doi.org/10.1016/S0966-842X(98)01408-5.

27. Swaminathan, G., Lin, S.-A., Patel, M., DiFelice, K., Smith, J., Gindy, M., Mahne, A., Cua, D., Casimiro, D., Espeseth, A., and Bett, A. (2017). Activation of the TLR2-MyD88 pathway is required for in-vivo efficacy of Lipid Nanoparticle based vaccine formulation. J. Immunol. *198*, 79.2. https://doi.org/10.4049/jimmunol.198.Supp.79.2.

28. Okamura, H., Kashiwamura, S., Tsutsui, H., Yoshimoto, T., and Nakanishi, K. (1998). Regulation of interferon-γ production by IL-12 and IL-18. Curr. Opin. Immunol. *10*, 259–264. https://doi.org/10.1016/s0952-7915(98)80163-5.

29. Ihim, S.A., Abubakar, S.D., Zian, Z., Sasaki, T., Saffarioun, M., Maleknia, S., and Azizi, G. (2022). Interleukin-18 cytokine in immunity, inflammation, and autoimmunity: Biological role in induction, regulation, and treatment. Front. Immunol. *13*, 919973. https://doi.org/10.3389/fimmu.2022.919973.

30. Sauer, J.D., Sotelo-Troha, K., Von Moltke, J., Monroe, K.M., Rae, C.S., Brubaker, S.W., Hyodo, M., Hayakawa, Y., Woodward, J.J., Portnoy, D.A., and Vance, R.E. (2011). The N-ethyl-N-nitrosourea-induced Goldenticket mouse mutant reveals an essential function of sting in the in vivo interferon response to Listeria monocytogenes and cyclic dinucleotides. Infect. Immun. *79*, 688–694. https://doi.org/10.1128/IAI.00999-10.

31. He, W.t., Wan, H., Hu, L., Chen, P., Wang, X., Huang, Z., Yang, Z.-H., Zhong, C.-Q., and Han, J. (2015). Gasdermin D is an executor of pyroptosis and required for interleukin-1β secretion. Cell. Res. *25*, 1285–1298. https://doi.org/10.1038/cr.2015.139.

32. Kim, B.S., Howell, M.D., Sun, K., Papp, K., Nasir, A., and Kuligowski, M.E.; INCB 18424-206 Study Investigators (2020). Treatment of atopic dermatitis with ruxolitinib cream (JAK1/JAK2 inhibitor) or triamcinolone cream. J. Allergy Clin. Immunol. *145*, 572–582. https://doi.org/10.1016/j.jaci.2019.08.042.

33. Verstovsek, S., Mesa, R.A., Livingston, R.A., Hu, W., and Mascarenhas, J. (2023). Ten years of treatment with ruxolitinib for myelofibrosis: a review of safety. J. Hematol. Oncol. *16*, 82. https://doi.org/10.1186/s13045-023-01471-z.

34. Ali, E., Owais, R., Sheikh, A., and Shaikh, A. (2022). Olumniant (Baricitinib) oral tablets: An insight into FDA-approved systemic treatment for Alopecia Areata. Ann. Med. Surg. *80*, 104157. https://doi.org/10.1016/j.amsu.2022.104157.

35. Urits, I., Israel, J., Hakobyan, H., Yusin, G., Lassiter, G., Fackler, N., Berger, A.A., Kassem, H., Kaye, A., and Viswanath, O. (2020). Baricitinib for the treatment of rheumatoid arthritis. Rheumatology *58*, 407–415. https://doi.org/10.5114/reum.2020.102006.

36. Tanaka, T., Narazaki, M., and Kishimoto, T. (2014). Il-6 in inflammation, Immunity, And disease. Cold Spring Harbor Perspect. Biol. *6*, a016295. https://doi.org/10.1101/cshperspect.a016295.

37. Chen, L., Zheng, L., Chen, P., and Liang, G. (2020). Myeloid Differentiation Primary Response Protein 88 (MyD88): The Central Hub of TLR/IL-1R Signaling. J. Med. Chem. *63*, 13316–13329. https://doi.org/10.1021/acs.jmedchem.0c00884.

38. Kumar, S.R.P., Biswas, M., Cao, D., Arisa, S., Muñoz-Melero, M., Lam, A.K., Piñeros, A.R., Kapur, R., Kaisho, T., Kaufman, R.J., et al. (2024). TLR9-independent CD8+

T cell responses in hepatic AAV gene transfer through IL-1R1-MyD88 signaling. Mol. Ther. *32*, 325–339. https://doi.org/10.1016/j.ymthe.2023.11.029.

39. Schroder, K., Hertzog, P.J., Ravasi, T., and Hume, D.A. (2004). Interferon-γ: an overview of signals, mechanisms and functions. J. Leukoc. Biol. *75*, 163–189. https://doi.org/10.1189/jlb.0603252.

40. Martino, A.T., Suzuki, M., Markusic, D.M., Zolotukhin, I., Ryals, R.C., Moghimi, B., Ertl, H.C.J., Muruve, D.A., Lee, B., and Herzog, R.W. (2011). The genome of self-complementary adeno-associated viral vectors increases Toll-like receptor 9–dependent innate immune responses in the liver. Blood *117*, 6459–6468. https://doi.org/10.1182/blood-2010-10-314518.

41. Levi, M., Schultz, M., and van der Poll, T. (2010). Disseminated Intravascular Coagulation in Infectious Disease. Semin. Thromb. Hemost. *36*, 367–377. https://doi.org/10.1055/s-0030-1254046.

42. Yang, X., Cheng, X., Tang, Y., Qiu, X., Wang, Z., Fu, G., Wu, J., Kang, H., Wang, J., Wang, H., et al. (2020). The role of type 1 interferons in coagulation induced by gram-negative bacteria. Blood *135*, 1087–1100. https://doi.org/10.1182/blood.2019002282.

43. Yang, M., Jiang, H., Ding, C., Zhang, L., Ding, N., Li, G., Zhang, F., Wang, J., Deng, L., Liu, J., and Xu, Y. (2023). STING activation in platelets aggravates septic thrombosis by enhancing platelet activation and granule secretion. Immunity *56*, 1013–1026.e6. https://doi.org/10.1016/j.immuni.2023.02.015.

44. Hsu, J.C.-C., Laurent-Rolle, M., and Cresswell, P. (2021). Translational regulation of viral RNA in the type I interferon response. Curr. Res. Virol. Sci. *2*, 100012. https://doi.org/10.1016/j.crviro.2021.100012.

45. Apelbaum, A., Yarden, G., Warszawski, S., Harari, D., and Schreiber, G. (2013). Type I Interferons Induce Apoptosis by Balancing cFLIP and Caspase-8 Independent of Death Ligands. Mol. Cell Biol. *33*, 800–814. https://doi.org/10.1128/MCB.01430-12.

46. Chawla-Sarkar, M., Lindner, D.J., Liu, Y.-F., Williams, B.R., Sen, G.C., Silverman, R.H., and Borden, E.C. (2003). Apoptosis and interferons: Role of interferon-stimulated genes as mediators of apoptosis. Apoptosis *8*, 237–249. https://doi.org/10.1023/A:1023668705040.

47. Shirley, J.L., de Jong, Y.P., Terhorst, C., and Herzog, R.W. (2020). Immune Responses to Viral Gene Therapy Vectors. Mol. Ther. *28*, 709–722. https://doi.org/10.1016/j.ymthe.2020.01.001.

48. Agudo, J., Ruzo, A., Kitur, K., Sachidanandam, R., Blander, J.M., and Brown, B.D. (2012). A TLR and Non-TLR Mediated Innate Response to Lentiviruses Restricts Hepatocyte Entry and Can be Ameliorated by Pharmacological Blockade. Mol. Ther. *20*, 2257–2267. https://doi.org/10.1038/mt.2012.150.

49. Shirley, J.L., Keeler, G.D., Sherman, A., Zolotukhin, I., Markusic, D.M., Hoffman, B.E., Morel, L.M., Wallet, M.A., Terhorst, C., and Herzog, R.W. (2020). Type I IFN Sensing by cDCs and CD4+ T Cell Help Are Both Requisite for Cross-Priming of AAV Capsid-Specific CD8+ T Cells. Mol. Ther. *28*, 758–770. https://doi.org/10.1016/j.ymthe.2019.11.011.

50. Monds, R., Miller, K., O'Hagan, A., Parsonnet, N., Cipi, J., Hanovice, N., Slubowski, C., Agosto, L., Lymar, A., and Stanton, M. (2024). iqDNA is an Engineered DNA Cargo That Avoids Innate Immune Activation While Retaining Durable Transgene Expression. Poster 1294 ASGCT Annual Meeting. May 11:2024.

51. Samuel, C., Cornman, H., Kambala, A., and Kwatra, S.G. (2023). A Review on the Safety of Using JAK Inhibitors in Dermatology: Clinical and Laboratory Monitoring. Dermatol. Ther. (Heidelb.) *13*, 729–749. https://doi.org/10.1007/s13555-023-00892-5.

52. Kato, H., and Kahlenberg, J.M. (2024). Emerging Biologic Therapies for Systemic Lupus Erythematosus. Curr. Opin. Rheumatol. *36*, 169–175. https://doi.org/10.1097/BOR.0000000000001003.

OMTN, Volume *36*

Supplemental information

Inhibition of IFNAR-JAK signaling enhances tolerability and transgene expression of systemic non-viral DNA delivery

Sujata Senapati, Thais B. Bertolini, Michael A. Minnier, Mustafa N. Yazicioglu, David M. Markusic, Rui Zhang, Joan Wicks, Ali Nahvi, Roland W. Herzog, Matthew C. Walsh, Pedro J. Cejas, and Sean M. Armour

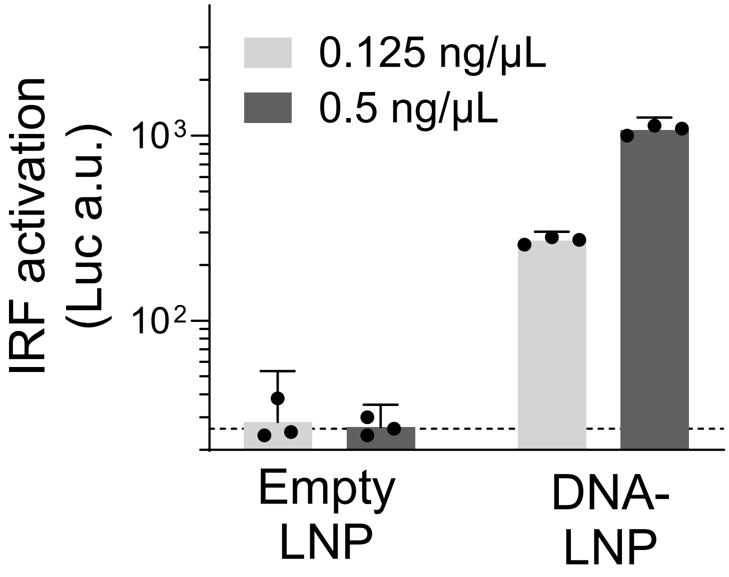

Figure S1. THP1-Dual cells were treated with either LNP encapsulating no payload (empty LNP) or plasmid DNA (DNA-LNP) at the indicated concentrations. IRF activation was measured by luminescence. Data shown as geomean with 95% CI; luminescence of untreated cells shown in dashed line.

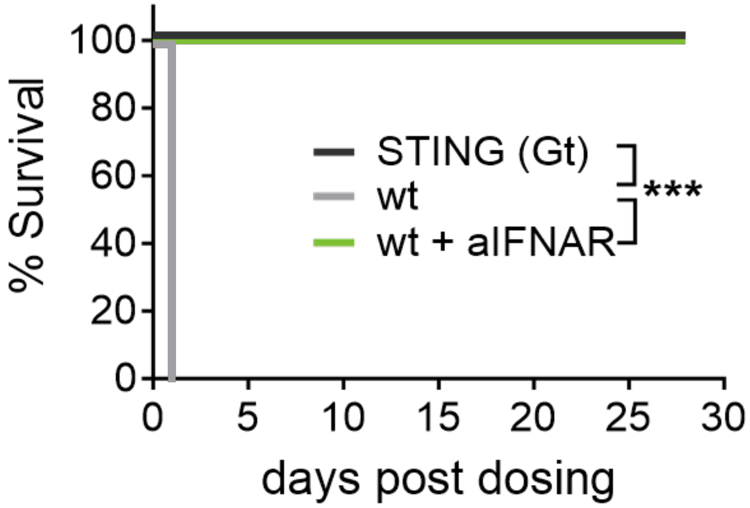

<u>Figure S2.</u> Wild-type (wt) and STING-deficient Goldenticket (Gt) mice (n=5 per group) were intravenously (tail vein) dosed with 100 µg of human Factor IX transgene encapsulated in LNP formulation (DNA-LNP). Wt mice were either untreated or treated with 300 µg per dose of anti-IFNAR intraperitoneally at 3 hours prior to DNA-LNP dosing. Survival of mice was followed out to 30 days post-dosing with of DNA-LNP. Survival data was analyzed by log-rank (Mantel-Cox) test; *** denotes $p<0.0001$
